# Technologies for Solubility, Dissolution and Permeation Enhancement of Natural Compounds

**DOI:** 10.3390/ph15060653

**Published:** 2022-05-25

**Authors:** Meshal Alshamrani, Muhammad Khalid Khan, Barkat Ali Khan, Ahmad Salawi, Yosif Almoshari

**Affiliations:** 1 Department of Pharmaceutics, College of Pharmacy, Jazan University, Jazan 45142, Saudi Arabia; malshamrani@jazanu.edu.sa (M.A.); asalawi@jazanu.edu.sa (A.S.); yalmoshari@jazanu.edu.sa (Y.A.); 2 Drug Delivery and Cosmetics Lab (DDCL), Faculty of Pharmacy, Gomal University, D. I. Khan 29050, Pakistan; barkat.khan@gu.edu.pk

**Keywords:** solubility, dissolution, permeation enhancement, natural compounds

## Abstract

The current review is based on the advancements in the field of natural therapeutic agents which could be utilized for a variety of biomedical applications and against various diseases and ailments. In addition, several obstacles have to be circumvented to achieve the desired therapeutic effectiveness, among which limited dissolution and/or solubility and permeability are included. To counteract these issues, several advancements in the field of natural therapeutic substances needed to be addressed. Therefore, in this review, the possible techniques for the dissolution/solubility and permeability improvements have been addressed which could enhance the dissolution and permeability up to several times. In addition, the conventional and modern isolation and purification techniques have been emphasized to achieve the isolation and purification of single or multiple therapeutic constituents with convenience and smarter approaches. Moreover, a brief overview of advanced natural compounds with multiple therapeutic effectiveness have also been anticipated. In brief, enough advancements have been carried out to achieve safe, effective and economic use of natural medicinal agents with improved stability, handling and storage.

## 1. Introduction

Natural products are the major sources for drug development because of diversity in their structures. They are isolated molecules obtained from plants, minerals or animal resources and used for a variety of therapeutic purposes of human and animal diseases [1]. Though the utilization of natural drugs is from ancient times, the drugs from natural sources still usually face drawbacks of numerous scientific evidences [1,2]. Despite this, natural products present the major source of biologically active molecules and play a main role in novel drug discovery [2]. Contrary to this, most natural products do not have characteristics of drugs and their pharmacological use is limited. Some of these characteristics lacking in natural products include low aqueous solubility, decreased dissolution rate, poor permeation and low absorption via biological membranes [2,3]. On the other hand, due to some severe adverse effects and appearance of resistance and/or recurrence in link with allopathic drugs, the research direction has been redirected towards the natural compounds [3]. On the contrary, the natural drugs are associated with enhanced safety, efficacy and with improved patient compliance [4]. In addition, some fields such as cancer-based research and treatments associated with allopathic drugs inclined a huge economic burden, but still, just the improvement in quality of life is ensured based on allopathic drugs [5].

So, based on the above-mentioned veracities, the natural compounds with several medicinal values have been addressed in the current review. It is factual that in the recent decades a lot of research and exploration has been carried out, but usually these studies and/or reviews do not fulfill the prerequisite of readership. The reason may attributed with the solitary or the title-specific direction of study(s) [6]. So, in the current review, efforts have been put forth to address multiple guidelines associated with natural compounds regarding their isolation and purification techniques. Likewise, a concise insight of natural compounds associated with their enhanced multiple therapeutic effects for cosmetics use and various other biomedical applications has also been portrayed in this review. In contrast, the major dispute associated with natural compounds which could be used on the basis of their therapeutic value is the poor dissolution/solubility and permeation of hydrophobic compounds and various strategies have been investigated to overcome these disputes [7,8].

## 2. Isolation and Purification of Natural Compounds

The isolation and purification could be taken as synonyms, so to avoid misleading and confusion, these two terms have been discussed in detail separately for the convenience regarding readership. The separate addressing of isolation and purification of natural compounds have been discussed below;

### 2.1. Isolation of Natural Compounds

The most important step associated with natural compounds is the isolation of these medicinal agents or compounds. In general, the isolation process could be categorized as; (a) old method for isolation of natural compounds or drugs and (b) new methods for isolation of natural compounds [9,10,11,12]. In old methods, limited studies were carried out including the; (i) selection of only those plant or plant parts which were known for their specific medicinal features, (ii) Identification of chemical nature of compounds and classification, identification or detection of compound(s) based on their chemical nature, (iii) Conventional method of isolation of natural compounds based on above-mentioned identification protocols, (iv) In vitro and in vivo characterization of these compounds, in which usually the in vivo characterization was carried out on human volunteers while the in vitro characterizations are carried out in laboratory set up, (v) Taxonomic classification of these compounds, (vi) Reporting of toxic characteristics of these compounds after experimentation/applications on humans [12,13].

**Scheme 1:**
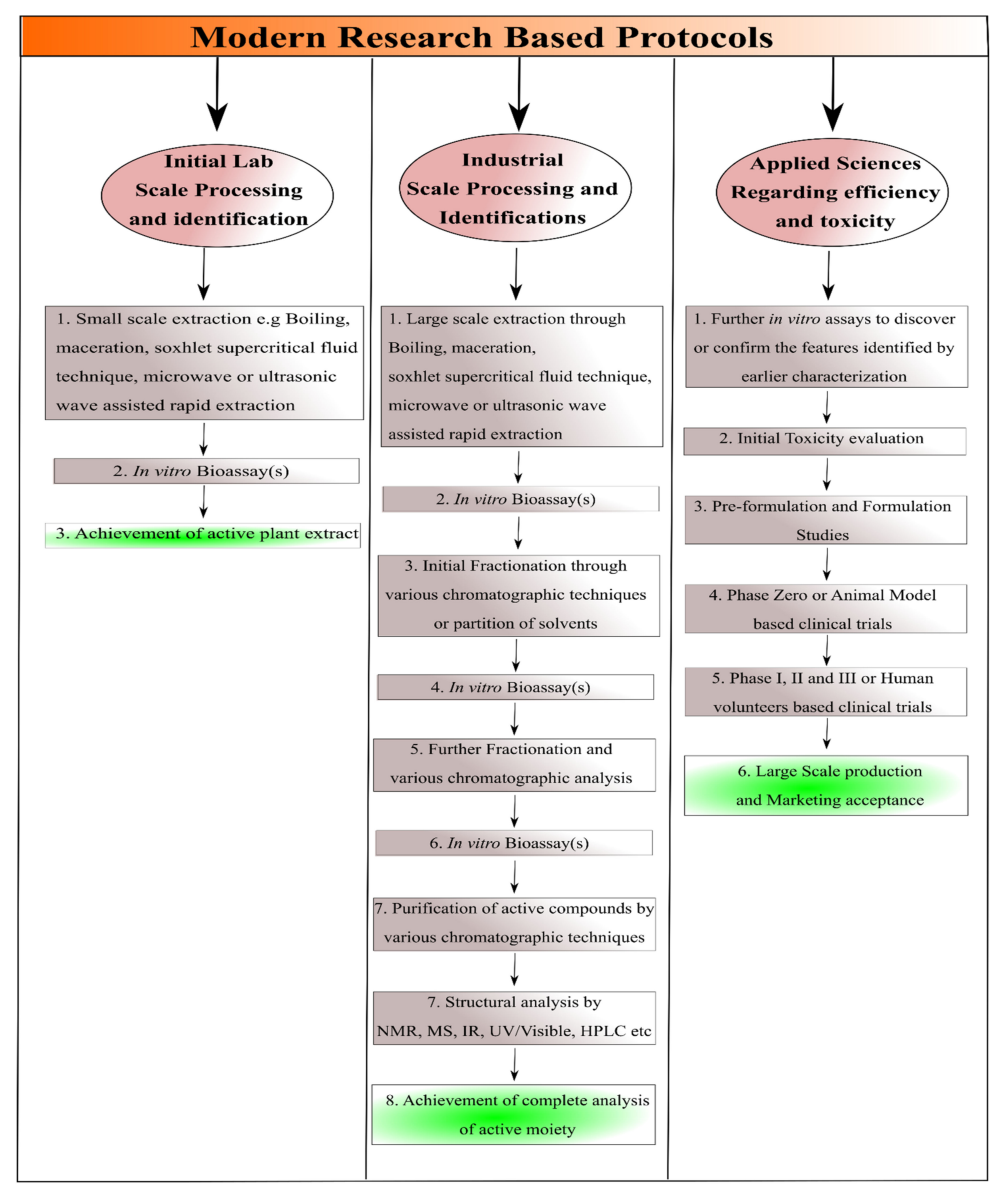
Dissolution and Permeation Enhancement of Isolated Purified Natural Compounds.

Before discussing the modern techniques for the isolation of natural compounds, it should be understood that in modern research, the protocols are carried out as reported by Sarker et al. (2005) and given in a Scheme 1 [14]. The modern technique regarding isolation of natural compounds may include; (a) In vitro bioassay-based characterization for the confirmation or final identification of the natural compounds after the achievement of small scale and large scale processing/evaluation parameters as illustrated in Scheme 1, (b) Achievement of such type of compounds in which further modification does not take place from the suitable evaluation parameters and comprehensive review of available literature, (c) Achievement of active moieties with medicinal effectiveness through advanced cell, tissue, genes or vector-based cultures either alone and/or via combinatorial strategy of these, (d) Achievement of sufficient data regarding the efficacy and toxicity profiling of natural compounds, (e) detection of their activity prior to the human-based clinical trials through various in vitro and in silico modeling approaches followed by ex vivo detection and subsequent in vivo animal-based clinical trials, (f) Combining the knowledge regarding selection of only those plant or plant parts which were known for their specific medicinal features (as mentioned earlier) and the modern techniques to achieve the best effective results, and (g) selection of other organisms or part of organism for the evaluation parameters such as marine algae, various bacteria and viruses, even in the modern scientific research cells, antigens or genes could be employed [8,9,10,11,12].

In addition, before initiation of isolation some of the features of natural compounds should be considered including water solubility, pH, size, charge and different types of stability profiles. Among these stability types, stability at different pH, temperatures, time periods and colloidal stability should be considered [15]. Das et al. (2021) comprehensively discussed the phytoecdysteroids and gathered the literature from 1999 to 2019 regarding methodology associated with the history, search for available literature, chemistry, new and old compounds isolated from phytoecdysteroids, mechanism of actions and their biomedical applications. The author reported that more than 400 types of phytoecsteroids have been reported from since 1954 up until now. The author reported 212 types of phytoecsteroids from eighteen families including amaranthaceae, asteraceae, blechnaceae, caryophyllaceae, clavicipitaceae, commelinaceae, cioscoreaceae, gleicheniaceae, lamiaceae, liliaceae, limnanthaceae, lygodiaceae, malvaceae, menispermaceae, polypodiaceae, polyporaceae, rhodomelaceae, and taxaceae [6]. The first phytoecsteroid called ecdysone was reported by Butenandt and Karlson in 1954 [16]. The common and most familiar isolated phytoecsteroids include 20-hydroxyecdysone, ponasterone A, ponasterone B, ponasterone C [6]. Likewise, Lafont et al. (1994) discussed the isolation of Phytoecsteroids in detail and reported that phytoecsteroids could be isolated through variety of protocols and/or techniques. Likewise, the isolation could also be achieved through reverse phase thin layer chromatography (RP-TLC), high performance liquid chromatography coupled with mass spectrometry (HPLC-MS), gas chromatography (GC), mass spectrometry (MS), gas chromatography coupled with mass spectrometry GC-MS, supercritical fluid chromatography (SFC) and supercritical fluid chromatography coupled with mass spectrometry (SFC-MS) [17]. For instance, Schooley et al. (1972) isolated phytoecdysone (phytoectosteroid) from *Podocarpus nakaii* leaves, by initially concentrating the extract containing 900 g of leaves with 80% ethanol, followed by dissolving in methanol and subsequent diluting with equivalent amounts of distilled water (750 mL). After that the impurities were removed by using hexane thrice and after achieving precipitate and aqueous layer, the aqueous layer was diluted with a sufficient quantity of water (4L). After that the filtrate was passed through the column and by varying the amounts of ethanol, finally three different types of the phytoecdysones (3% in total), namely ponasterone A, ponasterone B and ponasterone C, were achieved [18]. On the contrary a comparatively advanced type of isolation of phytoectosteroid, which is also an antimicrobial agent from *Nocardia levis* has been reported by Kavitha et al. (2010). The author discussed the isolation by the protocol initiated with culturing in a suitable medium followed by fermentation of broth, filtration by centrifugation and subsequent withdrawal of supernatant as filtrate. After that, the extraction was done with ethyl acetate and then the extract was concentrated through rotary evaporation. After that, the achieved dark brown was concentrated through column chromatography and then one of the fractions was carefully selected followed by structure interpretation through ultra-violet (UV), infra-red (IR) spectroscopies, mass spectrometry and nuclear magnetic resonance (NMR) spectrometry. After that, the successful isolation of 1-phenylbut-3-ene-2-ol was achieved [19]. In another study, Konishi et al. (1989) reported the isolation of antifungal cispentacin from bacillus cereus by a similar method with few modifications. In this method, after carrying out the initial three steps, column chromatography was achieved. The crude cispentacin was achieved, which was recrystallized with a mixture of acetone, ethanol and water and finally the pure form of cispentacin was achieved [20]. Among the recent studies, Wang et al. (2017) reported the isolation of Phytoecdysteroids from *Ajuga iva* for their anti-diabetic activity act. The isolation was carried out by drying 100 g of *Ajuga iva* leaves followed by extraction through distilled water via refluxing for 36 h at 90 °C, and subsequently, the conversion into powder by vacuum evaporation [21].

Guo et al. (2009) reported the isolation of two Bis(indolyl)methane alkaloids, namely streptindole and vibrindole A [22], whereas Abe et al. (2013) isolated five different types of Bis(indolyl)methane alkaloids including streptindole, vibrindole A, arsindoline A, arsindoline B and arundine [23]. Moreover, the author reported ten different types of isolation techniques associated with streptindole, two different types of isolation techniques associated with trisindoline, seven different techniques of isolation regarding vibrindole A, one isolation technique related to 2,2-bis(6-bromo-1 H-indol-3-yl)ethylamine and arundine and three isolation techniques linked with arsindoline A and arsindoline B. Regarding alkaloids, Praveen et al. (2015) gathered the data associated with the isolation of seven different types of Bis(indolyl)methane alkaloids in the review including streptindole, trisindoline, vibrindole A, 2,2-bis(6-bromo-1 H-indol-3-yl)ethylamine, arsindoline A and arsindoline B, arundine. Among these, most of the reported literature was based on the isolation of a single type of Bis (indolyl) methane alkaloids [24].

Likewise, Soares et al. (2015) reported the isolation of *Bocageopsis pleiosperma*-based alkaloids from the research-based study. In brief, initially the trunk bark, twigs and leaves were used and partitioned for the extraction of alkaloids for spectroscopic analysis (MS) and large-scale extraction, respectively. Regarding extraction, the samples associated with MS were dried, powdered, and stirred with aqueous solution of ammonium hydroxide and dichloromethane (DCM) followed by the transferring of organic phase in another container and subsequent stirring with acetic acid aqueous solution. On the contrary, the aqueous phase was separately treated with ammonium hydroxide to adjust the pH to 10, stirred with DCM and dried with anhydrous sodium sulfate and solvent evaporation in conjunction with nitrogen gas to achieve alkaloid. On the contrary, the large scale extraction was done by initially drying the leaves and conversion into powder followed by treating with ammonium hydroxide and DCM for 3 days. The organic and aqueous phase were separated such that the organic phase was agitated with aqueous acetic solution, whereas the aqueous phase was extracted with ammonium hydroxide, DCM and adjustment of pH 10 was carried out. Finally, the DCM-based phase was dried first with sodium sulfate and then evaporation to achieve the desired alkaloids. After that the fractionation and spectroscopic analysis were carried out for both samples i.e., sample associated with MS spectroscopy and large scale extraction [25]. Kukula-Koch et al. (2016) reported isolation of eight phenolic acids and two alkaloids as flavonoids from herb of *Berberis sibirica* by maceration and accelerated solvent extraction followed by identification and fractionation through various spectroscopic and chromatographic analysis. The spectroscopic analysis was carried out by Ultra High performance liquid chromatography (UHPLC), Hydrostatic counter-current partition chromatography (HCCC), High performance liquid chromatography with UV detector (RP-HPLC–DAD) and Ultra High performance liquid chromatography coupled with UV-detector, electrospray ionization and high resolution mass spectrometry UHPLC–DAD-ESI(±)HRMS techniques [26]. Likewise, Rinaldi et al. (2017), reported the isolation of four aporphine alkaloids, namely actinodaphnine, anonaine, isoboldine and nornuciferine from the extract associated with stem of *Annona hypoglauca*. The extraction was carried out by initial fractionation by column chromatography followed by elution through solvent mixture of DCM and methanol using different ratios to achieve 10 fractions. Among these three, fractions were again fractionated with preparative thin layer chromatography (PTLC) and after that actinodaphnine and nornuciferine were isolated by PTLC, whereas anonaine and isoboldine were isolated by HPLC. All these aforementioned compounds were then identified with HPLC [27]. Regarding terpenoids Rodrigues et al. (2009) and Skalicka et al. (2013) both reported the isolation of terpenoids by high-performance counter-current chromatography (HPCCC). In both these studies, sample preparation, selection of suitable solvent system and appropriate spectroscopic analysis was carried out by NMR, MS and finally with HPCCC. The difference was associated with selection of plant and plant part as Rodrigues et al. (2009) selected the stem of *Trichiliaquadrijuga*, whereas Skalicka et al. (2013) used Fruits of *Pimpinella anisum* for the extraction of terpenoids [28,29].

Among the recent studies, Kishore et al. (2018) reported the comprehensive study regarding isolation of a new compound called myricetin 3-O-(2″,4″-di-O-acetyl)-α-L-rhamnopyranoside and eleven known compounds from *Myrsine africana*. The known compounds were associated with flavonoids and flavonoids glycoside class of natural compounds including Myrsinoside A and B, quercetin, quercetin 3-O-α-L-rhamnopyranoside, quercetin 3-(3″,4″-di-O-acetyl)-α-L-rhamnoside, mearnsitrin and mearnsetin 3-O-(4″-O-acetyl)-α-L-rhamnopyranoside, myricetin 3-O-(4″-O-acetyl)-α-L-rhamnopyranoside, myricetin-3-O-(2″,4″-di-O-acetyl)-α-L-rhamnopyranoside, myricetin 3-O-α-L-rhamnopyranoside and rutin [30]. Likewise, based on the recent study associated with terpenoids, Zhang et al. (2021) reported the isolation of austrobuxusin-N, austrobuxusin A, austrobuxusin B, austrobuxusin C and austrobuxusin-D from the leaves of the *Austrobuxus swainii*. The author reported that isolation and identification and elucidation of structure was carried out through one dimensional nuclear magnetic resonance spectrometry (1D-NMR), two dimensional nuclear magnetic resonance spectrometry (2D-NMR) and mass spectrometry (MS). The 1D-NMR may also detect the peaks associated with functional groups but to avoid misleading associated with overlapping of peaks if any 2D-NMR was also used in conjunction which has the ability to differentiate overlapping peaks of functional groups as well [31]. 

### 2.2. Purification of Natural Compounds

As the isolated natural compounds may be associated with the presence of different types of impurities, so the natural compounds must be purified before carrying on pre-formulation and formulation processing and evaluations [32]. Therefore, the purification methods should be addressed so that the researchers seeking expertise in the isolation and extraction of natural compounds for various biomedical application should benefit. In addition, such data may also be added in the existing scientific knowledge for the wide community of readers. In this regard, a study conducted by Otsuka (2006) reported in a book chapter that purification associated with the achievement of desired natural compounds could also be achieved by the phenomenon of partition co-efficient. The author reported that the achievement of desired natural compounds could be achieved by partitioning the desired natural compound in immiscible solvents as mentioned earlier by Schooley et al. (1972) [18,33]. The author stated that it is comparatively easy compared to the achievement of desired natural compounds in the mixture of miscible solvents. In this technique the initial dissolving of extract in methanol followed by extraction with n-hexane are similar. However, after that, the dilution of methanol is carried with water to achieve different solutions (% *v*/*v*) i.e., 80% methanol-aqueous solution followed by extraction with CCl_4_ and 65% methanol-aqueous solution followed by extraction with CHCl_3_. By doing this, glycosides and hydrophilic polysaccharides could be achieved [33]. Likewise, Sulkowski (1985), in review, reported the purification of several types of proteins including Human serum proteins, Lactoferrin, α_2_-SH glycoprotein, Human fibroblast interferon, α_2_-Macroglobulin, Plasminogen activator, Lysozyme, Nucleoside diphosphatase, *Dolichos biflorus* lectin, Non-histone proteins, Human serum albumin, Human fibrinogen, Phosphotylrosyl-protein phosphatase Superoxide dismutase Human serum proteins, Bovine pancreatic ribonuclease, Cytochrome c, Calmodulin, Avidin, Myoglobins, Protein A, Albumins and Interferons by immobilized metal-affinity chromatography [34]. Later on in 1994, Chase reported the purification of proteins by adsorption chromatography [35]. Likewise, Dr. Granier reported the purification of proteins by electrophoresis [36]. In addition, Safarik and Mirka Safarikova (2004) in their review reported several types of proteins and peptides comprehensively, that may be purified through magnetic techniques. These proteins include aminopeptidases, ACE, caspase chymotrypsin, nuclear inclusion-a-proteases, bacterial trypsin, urokinases, lysozymes, various hydrolytic enzymes of carbohydrates and some other types of enzymes, antibodies, nucleotides or aptamer binding proteins, albumin and Hb and various other proteins [37]. In addition, similar to the polysaccharide purification the proteins may also be purified through a) denaturing of proteins followed by conversion of proteins to gel or jelly form, which can be purified or separated through centrifugation, and precipitation of proteins through Trichloroacetic acid solution or Trichloro ethane/Trifluoro ethane solution. The difference is that rather than using the supernatant, the supernatant will be used for the achievement or purification of proteins [38,39]. Moreover, the proteins could also be purified by salting out, PEGylation, dialysis, centrifugation, Lyophilization and ultrafiltration as reported elsewhere [39]. Recently, Eivazzadeh-Keihan et al. (2021), comprehensively described the purification of proteins and peptides by surface modification of nanoparticles with metals, polymers, biomolecules or antibodies [40].

Among lipids purification techniques, lipids could be purified through chloroform-methanol solvent system [41], by centrifugal partition chromatography [42,43], through supercritical fluids [44] and through membrane techniques [45]. The recent study is based on the purification of lipids by various techniques associated with lipid-based drug delivery approaches [46].

## 3. Physicochemical Characteristics Based Approaches for Solubility Enhancement

Normally, the extracted natural compounds are associated with low water solubility which may be challenging for the ideal bioavailability and subsequently optimum therapeutic effects. So variety of process variables are optimized to improve the solubility of these compounds and ultimately to achieve the desired therapeutic effectiveness [47]. In general, the solubility of the natural products may be enhanced by; (a) Particle size reduction, (b) Solid dispersion, (c) Complex Formation, (d) Natural products based Nanotechnology, (e) Micellar solubilisation and (f) Prodrugs [8] as illustrated in table (Table 1) and text below.

### 3.1. Particle Size Reduction

The particle sizes of natural compounds are reduced through various techniques, which may enhance the surface area. An increase in surface area may ultimately result in the improvement of aqueous solubility [67]. In general, the particle size may be reduced by comminution through grinding and/or jet milling [68], spray drying and recrystallization using anti-solvents through natural lipophilic compounds [69]. Charoenchaitrakool et al. (2000) reported the solubility enhancement of pure racemic enantiomer of ibuprofen and stated that 60% improvement in solubility was achieved through micronization associated with quick Expansion of Supercritical Solutions [70]. Likewise, Sievers et al. (2003) reported the solubility enhancement of natural and allopathic drugs and reported that micronization may enable the drugs suitable for pulmonary delivery [71]. In another study Suo et al. (2005) revealed the solubility enhancement of natural pigment called Bixin by particle size reduction through Pre-filming Atomization process [72]. In another study the author reported the solubility enhancement of salicylic acid and taxol by particle size reduction through supercritical solution expansion. The author also stated that the micronization and temperature both were inversely proportional to the particle size. In addition, it has also been reported that the method did not affect the chemical nature of compounds as well [48]. Likewise, the recent study reported by karimi and Raofie is based on the improvement of dissolution and water solubility by particle size reduction [73]. The authors reported the extraction of vincristine as vinca alkaloids which can be utilized for a variety of cancers from *Catharanthus roseus*. In this study, the solubility was enhanced by supercritical fluid expansion-based particle size reduction. 

### 3.2. Solid Dispersion

The solid dispersion is another auspicious strategy regarding the improvement of water solubility of drugs. It has been reported by Bikiaris in the comprehensive review that better enhancement of dissolution or solubility is achieved through solid dispersion compared to the micronization or particle size reduction. The author reported various preparation techniques of solid dispersions but primarily discussed the allopathic drugs as model drugs and marketed products associated with the solid dispersions of these allopathic drugs. The author discussed that possible techniques for the achievement of solid dispersion may include drug and carrier dissolution in organic solvent(s) followed by solvent evaporation, kneading technique, wet milling and spray drying [49]. Likewise, Vasconcelos et al. (2007) categorized the solid dispersions associated with allopatic drugs in three different generations as first, second and third generation cyclodextrins in the review. Vasconcelos et al. (2007) also reported that solid dispersion could be preferred over various other dissolution enhancement techniques due to their improved features in conjunction with the merits of solid dispersion [74]. Mishra et al. (2015) also highlighted the fourth generation of solid dispersions associated with allopathic drugs in addition to first, second and third [75]. Moreover, the author also reported various other techniques in addition to those discussed by Bikiaris (Solvent evaporation through vacuum drying and rotavapour, cryogenic processing, supercritical fluid technology, electrospinning, lyophilization and microwave irradiation). In addition, the author also discussed some limitations associated with solid dispersions in juxtaposition to its merits. Regarding natural medicinal agents, Zhaojie et al. (2014) reported the achievement of solid dispersion of natural alkaloid berberin in conjunction with permeation enhancer by solvent evaporation technique [76]. The author stated that the improved solubility and permeability were achieved which collectively enhanced the bioavailability of berberine. The author also publicized that it was the first study about berberine regarding its solubility and permeability enhancement and furthermore revealed that enhanced in vivo efficacy for the treatment of diabetes mellitus was achieved through the developed solid dispersion of berberine. Likewise, Zhang et al. (2014) also highlighted both in vitro and in vivo solubility enhancement associated with berberin-based solid dispersion. In the current above-mentioned study, both solid dispersion and complexation techniques were combined to achieve the synergistic solubility enhancement of berberin. The author reported that highest in vitro dissolution was achieved through the complexed solid dispersion followed by berberin solid dispersion and the least dissolution was observed through berberin [50]. In another similar study, Shi et al. (2015) presented the in vitro and in vivo evaluation of solid dispersion of berberine used in conjunction with hydrogenated soy phosphatidylcholine through vacuum desiccation and solvent evaporation technique. The author stated that an enhanced in vitro dissolution and in vivo bioavailability were achieved in the formulation of solid dispersion containing equivalent amounts of berberine and hydrogenated soy phosphatidylcholine [77]. Regarding glycosides, Pang et al. (2015) reported the solubility enhancement of rebaudioside D (belong to the class of steviol glycoside) and potassium sorbate (as a carrier)-based solid dispersion and stated that the solid dispersion was achieved through spray drying. The author revealed that the optimum enhancement in solubility was achieved through using rebaudioside D and potassium sorbate in equivalent quantities [78]. Kanaze et al. (2006) reported the development of solid dispersions using five flavonoids as flavanone glycosides, naringin, hesperidin, naringenin and hesperetin by solvent evaporation method. The author stated that two different sets were developed based on the carrier using polyvinylpyrrolidone (PVP) and polyethylene glycol (PEG) as carriers and revealed that PVP-based solid dispersions were completely amorphous and represented higher dissolution compared to PEG-based solid dispersions [79]. Likewise, Li et al. (2013) also reported the conversion of naringenin to amorphous form followed by solubility enhancement. The author used four different types of carrier including carboxymethylcellulose acetate butyrate (CMCAB), hydroxypropylmethylcellulose acetate succinate (HPMCAS), cellulose acetate adipate propionate (CAAP) and polyvinylpyrrolidone (PVP) and reported that the highest solubility was achieved through PVP-based solid dispersion and improved the water solubility of naringenin from 38 µg/mL to 8000 µg/mL [80]. Similarly Khan et al. (2015) reported the use of five different carriers and stated that kneading and rotary evaporation was carried out for the achievement of solid dispersion. The author reported the highest in vitro dissolution, solubility and in vivo bioavailability through soluplus^®^-based formulations. In addition, it was also highlighted that the solid dispersions achieved through solvent evaporation method were more soluble and with improved dissolution compared to the solid dispersions achieved through kneading technique [81]. Among the recent studies, Thenmzohie and Yoo (2017) reported the solubility enhancement of piperin from *piper nigrum* by solid dispersion through the use of hydrophilic carriers including sorbitol, PVP and PEG. The author stated the achievement of quick release of piperin through dissolution enhancement through these carriers. Less than 5% of pure piperine was released within two hours, whereas, more than 70% of piperine was released achieved through the aforementioned hydrophilic carriers-based solid dispersion of piperine [82].

### 3.3. Complex Formation

Complex formation is another promising approach regarding solubility enhancement of natural compounds. Among different type of complexations, the cyclodextrins (α-cyclodextrins, β-cyclodextrins and γ-cyclodextrins) gain sufficient scientific interest for solubility enhancement of pharmaceutical and cosmetic ingredients [51]. Uekama et al. (1983) initially diverted the research interest towards solubility enhancement of cardiac glycosides including digoxin, digitoxin and methyl digoxin through the aforementioned types of cyclodextrins (α-cyclodextrins, β-cyclodextrins and γ-cyclodextrins). The author mentioned the study outcomes and stated that the peak solubility enhancement was achieved through γ-cyclodextrins followed by solubility enhancement of β-cyclodextrins and the least solubility enhancement was achieved through α-cyclodextrins (improvement of the oral bioavailability of digitalis glycosides by cyclodextrin complexation) [51]. Likewise, in the last decade, Upreti et al. (2011) reported the complexation of α-cyclodextrins with steviol glycosides rebaudioside as rebaudioside A, rebaudioside B, rebaudioside C, rebaudioside D. rebaudioside E and rebaudioside F. The author stated that the solubility enhancement of the α-cyclodextrins complexed was achieved. In addition, the author reported that the effect was very prominent in rebaudioside A, rebaudioside B and rebaudioside D compounds as the solubility enhancement was directly proportional to the amounts of α-cyclodextrins as inclusion complexes [83]. Likewise, Wan et al. (2013) reported the solubility enhancement of resveratrol (as natural antioxidant) by complexation with another type of hydrophilic Steviol Glycosides known as stevioside. The author revealed that by increasing the quantity of stevioside, the solubility of stevioside resveratrol complex increases [84]. Zhang et al. (2016) reported the poor use of water soluble triterpenoid as a natural anticancer agents and reported the solubility enhancement through complexation with another type of highly water soluble steviol glycoside termed as rubusoside. The author reported that the optimum anticancer efficacy was not achieved from aforementioned triterpenoids in the previous studies due to its poor water solubility and contrarily improved and optimum anticancer efficacy against Caco-2 cells was achieved after complexation with rubusoside [52]. Ping et al. (2017) reported the 4.3 times higher solubility of sanguinarine after complexation with highly water soluble carboxylatopillar-6-arene which is among the class of natural compounds called pillararenes. The sanguinarine was used as a model natural medicinal agent belong to the class of alkaloids and is indicated for various types of microbial infections, inflammations and cervical cancer [85]. Nguyen et al. (2017) used four different types of steviol glycosides for complexation of epigallocatechin gallate namely rubusoside, stevioside, rebaudioside A and stevioside glucosides. The author reported improved antibacterial activity, antioxidant activity and 16- to 17.5-fold enhanced solubility achievement through the complexes. Furthermore, among the aforementioned four complexes highest water solubility was achieved through the epigallocatechin gallate-stevioside complex [86]. The recent studies are associated with the in silico modeling of tropane alkaloids and beta cyclodextrins complex through Molecular dynamic simulation and docking study. The author stated that directly proportional behavior of solubility and beta cyclodextrins was observed. Analytical and in silico study was of the inclusion complexes between tropane alkaloids atropine and scopolamine with cyclodextrins [87]. The most recent study by Ko et al. (2021) also highlighted the use of steviol glycosides for the conversion of poorly water soluble curcumin into water soluble curcuminoids (Curcumin-steviol glycosides complex) for antiviral theapy. Moreover, the author reported the novel method of Microwave-assisted extraction and stated that by using in conjunction with the Curcumin-steviol glycosides complex several folds enhanced solubility (11 mg/L to 1320 mg/L) was achieved [53].

### 3.4. Natural Products Based Nanotechnology

Regarding solubility enhancement associated with the natural medicinal agents based nanotechnology, an enormous research work has been carried out. Therefore, a huge literature is available in this esteem and it is difficult to congregate the whole literature. Therefore, in this review a number of review articles have been discussed which in turn contain a colossal literature associated with the solubility enhancement of natural medicinal agents through nanotechnology. In general, it is scientifically discussed by various researchers that by decreasing the size of the drug delivery vehicle to the nanosized range, it provides improved solubility and targeted drug delivery. In this instance, Mirhadi et al. (2018) discussed the possible techniques for solubility enhancement associated with berberine (a natural alkaloid) through the review of an extensive literature in the review article [88]. Likewise, Ahmad et al. (2018) comprehensively discussed the solubility enhancement and other challenges with solutions for the cosmetic application of natural medicinal agents. In addition, the author reported different types of nanotechnology-based approaches in the review [54]. Qiao et al. (2020) reported the nanotechnology-based drug delivery and solubility enhancement of traditional Chinese medicines in the review. Furthermore the author discussed variety of traditional Chinese medicines and various nanotechnology-based drug delivery approaches in the review [89]. Teng et al. (2021) painstakingly discussed variety of nanotechnology based drug delivery approaches for the bioavailability enhancement of flavonoids derived from food products or diet. In the review, the author discussed various types of nanotechnology-based drug delivery approaches for the enhancement of solubility, permeability and targeted drug delivery [55]. The recent study is based on the enhanced delivery of medicinal agents extracted from various plants through various nanotechnology based drug delivery approaches. The author reported the successful achievement of solubility enhancement and permeability enhancement through various nanotechnology based drug delivery vehicles [56]. Moreover, McClements and Öztürk (2021) revealed the proper handling and storage of medicinal lipids through various nanotechnology-based drug delivery approaches to achieve optimum therapeutic effects [12].

### 3.5. Micellar Solubilization

The micellar solubilisation is another most explored area of research for the solubility enhancement of both natural as well as allopathic drugs. It is very difficult to assemble all the research and literature associated with micellar solubilization. Therefore, the recent research work has been highlighted regarding micellar solubilization. In this regard, Wang et al. (2016) reported the use of myrcetin with limited passage across blood brain barrier (BBB) and aimed to deliver it to the brain. The author used sodium dodecyl sulphate and Pluronic F68 as carriers and Labrasol as a surfactant to achieve myrcetin-based micelles, such that the micelles were achieved through solvent evaporation method. The developed micelles showed pH-dependent enhanced dissolution, drug release and in vivo studies depicted enhanced bioavailability and delivery of myrcetin to the brain [90]. Likewise, Khayyal et al. (2018) stated the use of curcumin and boswelia which are indicated for anti-inflammatory effects, but their use is limited due to their low water solubility. So in this study it has been reported that the enhanced in vitro dissolution and solubility and better in vivo anti-inflammatory effects were achieved [91]. Likewise, Śliwa et al. (2019) reported the use of Rokanols^®^ as non-ionic surfactants for the micellar solubilization. It has been deducted that both molecular dynamic simulation and experimental studies revealed an enhanced solubility of Rokanols^®^-based narcissin (flavonoid) micelles compared to the pure narcissin [57]. Khonkarn et al. (2020) reported the use and loading of three flavonoids, namely quercetin, quercetrin, and rutin in the conjugated polymeric micelles for an anticancer efficacy. The author stated that all these flavonoids are poorly water soluble and micelles caused significant improvement in the dissolution of flavonoids. Among these flavonoids rutin-based polymeric micelles showed highest dissolution and drug release. Moreover, the in vitro cytotoxicity study revealed an enhanced anticancer efficacy against sensitive and multi drug resistance-based leukemia and the conjugated polymer was found to have resistance reversing properties as well [92]. Regarding glycosides, Zhou et al. (2021) reported the solubility enhancement of vitamins (vitamin D2 and Vitamin E) by rebaudioside A, which is stevioside glycoside. The author reported that rebaudioside A promoted micellar slubilization of above mentioned vitamins and enhances the solubility of vitamins several hundreds to several thousand folds by rebaudioside A through micellar solubilization [93]. The recent study by Tiwari et al. (2022) also highlighted the enhanced solubility of quercetin by micellar solubilization. The author used Tetronic^®^ T904 as block copolymer which caused efficient micellar solubilization. The author also revealed that an increase in size of micelles increased the solubility of quercetin and vice versa [94].

### 3.6. Prodrugs

Prodrugs is based on another efficient strategy regarding solubility enhancement and received enough scientific attention. Various types of prodrugs have been developed to facilitate efficient enhancement in dissolution and solubility of natural products. For instance, Kim et al. (2009) reported the conversion of quercetin (3′,4′, 4, 5, 7-pentahydroxyflavone) which is a natural flavonoids, into prodrug as 3-n-n-dimethyl carbamoyl Quercetin. The author stated that by doing so, the solubility and anticancer efficacy both were improved as this compound was resistant to hydrolysis in the medium, where culture of cells is carried out [95]. Liu et al. (2016) reported the use of natural compound associated with Chinese traditional medicine termed as bufalin. Bufalin is associated with anticancer effects, but due to lower solubility, limited anticancer effects could be achieved. So in this study the prodrug approach through PEGylation and carboxylic acid conjugation was used which enhanced the solubility of bufalin, which in turn, increases the anticancer efficacy of bufalin as well [96]. Chen et al. (2018) reported the use of 7,8-dihydroxyflavone-based prodrug which has the ability to treat Alzheimer’s disorder. Due to its limited solubility, bioavailability and passage across BBB, the optimum therapeutic effect against Alzheimer’s diseases could not be achieved. Therefore, the author used the prodrug approach to enhance the solubility and passage across BBB. The author stated that both in vitro and in vivo studies highlighted the optimum delivery of prodrugs against Alzheimer’s disease [97]. Zhou et al. (2017) comprehensively discussed the triterpenoids-based prodrugs for the enhancement of solubility and targeted delivery in the review. The author described few natural compounds and stated the several hundred time enhanced solubility could be achieved through prodrugs based drug delivery approach [98]. Li et al. (2020) reported the use of specific cardiac glycoside (TXA9), known for its anticancer efficacy, with poor solubility, which limited its use. For this purpose, Li et al. developed TXA9-based polymer prodrug using polyethylene glycol (PEG), glycin and α-Tocopherol polyethylene glycol succinate (TPGS) conjugated TXA9. The author revealed that enhanced solubility and improved anticancer efficacy of above stated cardiac glycoside against lung cancer was achieved [99]. Among the recent studies, Abouaitah et al. (2020) reported the achievement of enhanced solubility and effective delivery to the cancer site of piperine prodrugs by loading in hydroxyapatite nanoparticles. Moreover, the author reported that pH-dependent solubility was observed, such that by decreasing the pH the solubility of piperine prodrugs increased. Such behavior also favors the delivery of piperine to the cancer site as tumor site is associated with slightly acidic pH due to the lactic acid accumulation [100]. Likewise, Omran et al. (2021) recently reported 80-times (approx.) enhanced solubility of Phenanthroindolizidines class of alkaloids (antofine and tylophorine) in the reported study. Moreover, the above mentioned natural compounds have low passage across BBB, but by conversion into prodrug efficient delivery to brain was established [58].

## 4. Permeability Enhancement

There are copious advancements regarding permeability enhancement and it is nearly impossible to deal with the whole research work carried out in this field. However, it could be possible to highlight the major technique for the permeability enhancement of natural medicinal agents. In general, the permeability could primarily be enhanced with the use of (a) permeation enhancers and (b) incorporation or encapsulation of natural compounds or agents within lipid based drug delivery systems. Regarding the permeation enhancers, Williams and Barry (2021) comprehensively discussed different types of penetration enhancers and the purpose for which it can be used [59]. The author stated that, in general, penetration enhancers can improve the permeability of natural compounds by; (i) Modification or denaturation of keratins followed by enhanced permeation of active moiety across the skin layers [60,101], (ii) Interaction with desmosomes which are generally involved in the maintenance of cohesion between corneocytes and affect the cohesion between corneocytes, followed by increases in the spaces between corneocytes and subsequent enhanced permeation of therapeutic agents [102], (iii) Disruption/modification of the lipid bilayer [103] and (iv) Modification of partition of natural medicinal agents by affecting the vehicles or fluids of stratum corneum, thus improving the permeation across the skin layer and/or absorption within the blood [104]. In addition, Chen et al. (2016) comprehensively reported that natural terpenes could itself act as permeation enhancers associated with enhanced permeation of natural medicinal agents as well as allopathic drugs [105].

Shishir et al. (2019) comprehensively described in their review articles the efficient permeability enhancement of natural products or medicinal agents by encapsulation in liposomes [61]. Entrapment of natural medicinal agents within lipid-based drug delivery systems also received sufficient scientific concern and it may also be considered as a well-reputed and highly explored area of research [62]. Likewise, Pardeike et al. (2009) and recently Saka and Chella (2021) also reported the efficient permeability enhancement of natural agents through encapsulation within solid lipid nanoparticles (SLNs) and nanostructured lipid carriers (NLCs) [63,106]. Even Puglia et al. (2019) revealed that SLNs and NLC may act as a perfect combination for natural medicinal agents regarding permeation improvement of natural medicinal agents [64].

## 5. Advanced Natural Products for Pharmaceutical and Cosmetics Use

The natural products provide an important foundation as potential lead compounds for the development of new and more effective drugs for pharmaceutical and cosmetics use through structural modification. Due to their diverse and complex chemical structures, the secondary metabolites of plants seem to exhibit greater biological friendliness and drug-likeness than those derived from purely synthetic sources. Consequently, the molecules from natural origin are considered to be better candidates for further drug development and discovery [65]. The advancement in the field of natural products as medicinal agents enabled the isolation of various natural compounds through a variety of isolation and purification techniques as stated earlier. Copious advancements associated with natural compounds enabled the researchers to explore advanced drug molecules and diverted the direction of researchers from allopathic drugs towards natural compounds over again [66]. Therefore, a specific focus needs to be drawn towards the advanced natural compounds regarding their enhanced capability for cosmetics and various other biomedical applications. So based on this phenomenon, some of the advanced natural compounds have been addressed in the table (Table 2) and text below;

### 5.1. Kojic Acid

Kojic acid is a microbial metabolite that could be obtained from various types of microbes including the genus of Aspergillus (fungi responsible for causing aspergillosis/fungal lung infection), penicillium (renown fungi belongs to ascomycetes) and acetobactor (bacteria that may convert ethanol into acetic acid) [65,66]. In 2001, Masse et al. reported that kojic acid could be used as skin whitening agent [66]. Likewise, in 2004, Huang et al. stated the effectiveness of kojic acid in conjunction with other natural compounds as skin whitening and cosmetic application [155]. Later on, in 2008, Rho et al. reported six different types of kojic acid derivatives containing two kojic acid molecules against melanocytes followed by achievement of improved skin whitening [156]. Likewise, In 2012, Higashi and Fujji, [157] and Lajis et al. [158], In 2015, Wang et al. [159], In 2016, Galimany-Rovira et al. [160], singh et al. [107], In 2019, Saeedi et al. [161] and, recently Lee et al. (2020) reported the anti-melanogenic and skin whitening potential of Kojic acid [162]. Likewise, the recent studies also highlighted an improved in vitro and in vivo anti-inflammatory and antioxidant activities associated with Kojic acid as well [111,163]

Furthermore, Wei et al. (2011) also reported that kojic acid derivative based complex may also be employed for their anti-diabetic activity [108]. Moreover, Karakaya et al. (2018) [109] and Annan et al. (2019) [164], reported that kojic acid may be employed for their anticancer efficacy against melanoma cells. Likewise, El-Korany et al. (2020) also pointed towards the mechanistic insight associated with the possible activity of Kojic acid against cardiovascular disorders by inhibiting pancreatic lipases [110]

### 5.2. Gallic Acid

The gallic acid belongs to the class of tannins also termed as Gallo-tannins [112]. Regarding cosmetic applications, similar to Kojic acid (as mentioned earlier), the gallic acid could also be employed as skin whitening agents and against hyperpigmentation [165]. In addition to that, it could also be employed as anti-aging and for anti-oxidant activity as reported by Kim (2007) [166], Manosroi et al. (2011) [113], Kumar et al. (2013) [114] and Nam et al. (2017) [167].

Moreover, Su et al. (2013) [168] and Verma et al. (2013) [169] also reported enhanced in vitro cytotoxicity, apoptosis, cancer cell uptake and anti-cancer efficacy of gallic acid as well. Furthermore Tsang et al. (2016) [116] and Soe et al. (2016) reported the improved anti-inflammatory effects of gallic acid [117], whereas, Liu et al. reported the dual anti-allergic and anti-inflammatory effects of gallic acid [170]. Besides this, Karimi-Khouzani et al. (2017) reported the capsaicin-based improved healing of impaired liver associated with in vivo experimental model of rats [120]. Farther, an antidiabetic activity associated with gallic acid has also been reported by Liu et al. (2013) [119], Fachriyah et al. (2017) [115] and Variya et al. (2020) [171]. The recent reported study revealed that gallic acid may also be used as cardio-tonic in conjunction with anti-oxidant and anticancer effects [172].

### 5.3. Capsaicin

Capsaicin is natural medicinal agent with enhanced tumoricidal effects and could be derived naturally from chili pepper [118]. For instance, Shin et al. (2008) reported enhanced in vitro cytotoxicity against human melanoma cancer cell line (B16-F10 melanoma) [173]. Likewise, Hong et al. (2018) [174] and Wu et al. (2020) also reported improved anti-cancer efficacy of capsaicin against melanoma [175]. In addition, Zhang et al. (2017) [123], Thongnum and Chanthai (2018) [176] and Sanati et al. (2018) [122] revealed improved antidiabetic activity of capsaicin as well. Moreover, Kim et al. (2013) [177], Lee et al. (2007) [178], and Jolayemi and Ojewole (2003) also publicized improved ant-inflammatory effects [179], whereas, Redington et al. (2012) stated that capsaicin may also be used as cardio-tonic [124].

Furthermore, Hassan et al. (2012) reported multiple biomedical applications including anticancer, antioxidant and hepato-protective functions associated with capsaicin [125]. Likewise, among the recent studies Zhang et al. (2019) [121] and Karimi-Sales et al. [180] also reported protective functions against damaged liver associate with capsaicin.

### 5.4. Omega 3 and Omega 6 Fatty Acids

Omega 3 and Omega 6 both are types of polyunsaturated fatty acids. Among these, omega 3 fatty acids are considered as good lipid and help in lowering the blood cholesterol levels [126]. In addition, Omega 6 fatty acids are primarily considered as the structural and functional influencers of cell membrane and as precursors of prostaglandins. The prostaglandins are basically involved in the functional activity of cyclooxygenase (COX) enzymes, which are involved in inflammation, pain sensitivity and pyrexia [181]. In 2011, Brignole-Baudouin et al. (2011) reported the omega 3 and omega 6 fatty acids against dry eye syndrome and the author reported the enhanced efficiency of omega 3 and omega 6 fatty acids regarding treatment of dry eye syndrome through controlling the inflammation. Such effect may be primarily associated with omega 6 fatty acids as mentioned earlier [182]. Likewise, Innes and Calder (2018) also stated the improved ant-inflammatory efficiency associated with omega 6 fatty acids [183]. Besides this, Ionescu et al. (2015) [130], Huang et al. (2018) [128] and Balić et al. (2020) [184] reported that omega 3 and omega 6 fatty acids could be used as anti-aging, as cosmetics and against various skin orders or healing of skin, whereas, Diwakar et al. (2014) reported the skin whitening effects associated with these fatty acids from *Punica granatum* [185]. Moreover, Johnson and Bradford (2014) reported that these fatty acids may also be used against cardiovascular disorders, which is in accordance with the efficiency of omega 3 fatty acids (as mentioned earlier) [127]. In addition, Chajès and Bougno (2003) reported the improved anticancer efficacy of omega 3 and omega 6 fatty acids against various types of cancers as well [131].

Moreover, Adili et al. (2018) also reported the mechanistic insight regarding platelet aggregation and wound healing associated with omega 3 and omega 6 fatty acids [129]. Furthermore, Lee et al. (2007) also reported that omega 3 and omega 6 fatty acids from different sources could be used against various liver diseases and/or disorders [186].

### 5.5. Genistein

Genistein is an isoflavone associated with phytoestrogens [132,187], so primarily it is considered to have role in human endocrinology and against various endocrine disorders as reported by Zhang et al. (2018) [188], Rajaei et al. (2019) [189] and Eustache et al. (2020) [133]. Besides that, genistein also has improved anti-aging and antioxidant activity [190] and could be used in cosmetics and against various skin disorder including bacterial acne as reported by Oh et al. (2020) [191].

In addition, it has been reported that genistein also has Hepato-protective function or healing of damaged liver in in vivo animal models as reported by Fan et al. (2015) [135] and Ganai and Husain (2017) [192]. Moreover, various research-based studies also revealed an enhanced anticancer efficacy associated with genistein against cervical cancer [137] and various other types of cancers [134,193]. In addition, an improvement in cardiovascular events and against various cardiovascular disorders in males as well as postmenopausal women in a chronological manner has been reported by Dixon and Ferreira (2002) [194], Altavilla et al. (2004) [195], Crisafulli et al. (2005) [196], Si and Liu (2007) [197], Marini et al. (2010) [198], Sureda et al. (2017) [199], Bai and Wang (2019) [200], Thangaval et al. (2019) [201], Amerizadeh et al. (2021) [202] and Ye et al. (2021) [136]. Likewise, anti-inflammatory effects associated with genistein have also been reported by Shahmohammadi et al. (2018) [203], Du et al. (2018) [204] and Liu et al. (2019) [205], respectively.

### 5.6. Carotenoids

Carotenoids are the naturally occurring lipophilic pigments that could be derived from microbes and plants and among carotenoids β-carotenoids are considered as the fat soluble provitamins associated with retinol (vitamin A) [206]. The carotenoids could be employed for cosmetics use and anti-oxidant properties as reported formerly by Terao (1989) [207] and Bayerl (2008) [208] and recently by Ge et al. (2015) [209], Joshi et al. (2018) [138], Morocho-Jácome et al. (2020) [210], Manowattana et al. (2020) [211] and Bin-Jumah et al. (2021) [212]. In addition, Machlin (1995) reported the anti-oxidant activity of carotenoids and in turn anticancer efficacy and against various cardiovascular disorders associated with anti-aging effects of carotenoids [213]. Moreover, the research-based studies also revealed that carotenoids may be employed against various types of cancers including liver cancer and colorectal cancer as reported by Wang et al. (2012) [214] and Lan et al. (2016) [215]. Furthermore, in recent years an antidiabetic activity of carotenoids has also been explored as reported by Biworo et al. (2015) [140], Yaribeygi et al. (2019) [216] and Nimbalkar et al. (2021) [217]. Besides these, Fuller et al. (2006) [139], Hernández-Ortega et al. (2012) [218] and Honarvar et al. (2017) [219] reported an improved ant-inflammatory activity associated with carotenoids as well. Among the recent studies, Elvira-Torales et al. (2019) comprehensively described the hepatoprotective activity associated with carotenoids [141].

### 5.7. Lupeol

Lupeol could be obtained from various sources and may be considered as the triterpenoid class of phytomedicines [142]. The Lupeol could be employed for various cosmetics and biomedical applications. For instance, Pérez-González et al. (2019) reported the anti-oxidant activity of Lupeol [220]. Likewise, Tiwari et al. (2019) also reported improved anti-oxidant or anti-aging features associated with Lupeol [221]. In addition, Lakshmi et al. (2014) highlighted an antidiabetic activity of Lupeol [143]. Moreover, Bhatt et al. (2021) reported an improved anticancer efficacy against various types of malignancies [144], whereas, Liu et al. (2021) [145] revealed dual anti-inflammatory and anticancer activities linked with Lupeol. Furthermore, Prasad et al. (2007) stated enhanced hepatoprotective activity associated with Lupeol against damaged liver [146]. In addition, Siddique et al. (2011) highlighted that Lupeol could also be employed against various endocrine disorders [147].

### 5.8. Colchicine

Colchicine is associated with the plant alkaloids usually extracted from *Colchicum autumnale* and is considered as collagen secretion inhibitors by inhibiting polymerization of microtubules required for collagen secretion. So colchicine may in turn act as antifibrotic and collagen synthesis inhibitor [148].

In brief, the colchicine has been used in various research-based studies as skin whitening and anti-oxidant or anti-aging activity regarding cosmetic application as reported by Goo et al. (2018) [222] and Bhuvneswari et al. (2020) [149], respectively. In addition, Wang et al. (2021) reported that colchicine could effectively be used against anti-diabetic activity [150]. Moreover, Gasparyan et al. (2015) [151] and Paré et al. (2020) [152] reported an enhanced anti-inflammatory activity associated with colchicine. Furthermore Casanova et al. (2015) stated that colchicine may also be used against various cardiovascular disorders [223] and recently Awad et al. (2022) revealed an enhanced healing of damaged liver through colchicine [153].

## 6. Challenges Associated with Natural Compounds

The biggest challenge is the achievement of suitable size of the desired active moiety from the lowest possible amounts of extract associated with its natural source as in most of the cases very limited yield of desired natural compounds is achieved from a huge quantity of extract. For instance, Farnsworth (1990) reported that for the isolation of 30 g of vincristine (natural anticancer agent), the dried leaves equivalent to 15,000 kg were obtained from *Vinca rosea* [154]. Likewise, Schooley et al. (1972) reported that from 900 g of *Podocarpus nakaii* dried leaves, only 6.65 g of phytoecsteroids were obtained (as mentioned earlier) [18]. Another challenge is the achievement of suitable size, as various techniques and equipment may be employed to achieve the optimum particle size of the natural products [224]. Additionally, the achievement of new chemical entity, as this case may undoubtedly be associated with a huge success, but for complete profiling and identification lots of experimentation, equipment and proper handling and storage is required as reported by Kishore et al. (2018) (mentioned earlier) [30]. Likewise, in some cases the solubility or permeability enhancers are used in conjunction with the active moiety which may also cause possible interactions among the constituents of formulations or with skin moieties [225]. In general, it is considered that the natural compounds are safe for health, but it is not true as these natural compounds may be very toxic. So, the estimation of the toxicity of the natural compounds is required, which is a perplexing and challenging task [226]. The natural compounds are not conceded through the human-based clinical trials, so the natural compounds may put question mark on their safety and efficacy [227]. Likewise, the natural source contains multiple natural compounds so it is difficult to achieve the desired compound in the amounts required to achieve optimum therapeutic effectiveness [228]. Moreover, some natural compounds such as cholesterol may interact with the encapsulation of moiety within nanoparticles [229,230]. In specific, the solubility enhancement associated with natural medicinal agents require the optimization regarding identification, isolation and purification procedure [231]. In addition, suitable handling, storage conditions, selection of suitable surfactants and/or carrier and/or block copolymers and process parameters are required. Likewise, the selection of appropriate dosage, frequency and route of administration are also important to recognize [231,232,233]. Regarding the permeability enhancement, a huge literature is available, but still the selection of appropriate permeation enhancer is an important parameter. The careful selection of permeation enhancer which is compatible with the natural active moiety should be selected. In addition, the permeation enhancers with least toxicity should be selected and should be in accordance with the objective of the research work [234]. In both cases of solubility, enhancement and permeability enhancement, the nanotechnology are considered as the smart and modern efficient drug delivery strategies. Nevertheless, it should be kept in mind that nanotechnology-based carriers or surfactants are not completely safe and they have some health-based concerns, so proper nanotechnology-based drug delivery approaches also perceive considerable importance. In addition, another huge challenge is the cost effectiveness associated with solubility and permeability enhancement as in the case of nanotechnology-based drug delivery systems the expensive carriers, surfactants and other excipients are required to achieve the suitable drug delivery systems [11].

## 7. Conclusions

It can be concluded from the data presented in this review that natural products are the major resources of novel drug discovery, however they face the various physicochemical drawbacks which are attempted to be overcome by the conventional as well as the advanced techniques. It is also summarized that natural products have been used for the treatment of various communicable and non-communicable diseases from ancient times and even today. The advancements in natural compounds associated with improved therapeutic efficacy is of very much importance and needs to be explored. So an effort is put forth to make sufficient data available to the wide community of readers regarding efficient isolation and purification techniques in conjunction with solubility and permeability enhancement of natural compounds for cosmetic use and various biomedical applications.

## Figures and Tables

**Table 1:** An insight into solubility and permeability enhancement of natural products isolated from single or multiple sources.

**Solubility Enhancement Techniques**					
**Serial No.**	**Techniques**	**Constituent(s)**	**Source**	**Family**	**Reference**
1.	Particle size reduction	Bixin	*Bixa orellana* L.	Bixaceae	[48]
		Vincristine	*Catharanthus roseus*	Apocynaceae	[49]
2.	Solid dispersion	Berberin	*Rhizoma coptidis*	Ranunculaceae	[50]
		Piperine	*Piper nigrum*	Piperaceae	[51]
3.	Complex formation	Resveratrol	*Vitis vinifera*	Vitaceae	[52]
		Atropine and scopolamine	*Datura stramonium* L.	Solanaceae	[53]
4.	Natural products based nanotechnology	Berberin	Multiple sources	Berberidaceae	[54]
		Traditional Chinese medicines	Multiple sources	Different families	[55]
		Multiple constituents (Flavonoids)	Multiple sources	Different families	[56]
5.	Micellar solubilization	Curcumin	*Curcuma longa*	Zingiberaceae	[57]
6.	Prodrugs	Piperine	*Piper nigrum*	Piperaceae	[58]
		Antofine and Tylophorine	Multiple sources	Asclepiadacea	[59]
**Permeability Enhancement Techniques**					
**Serial No.**	**Techniques**	**Constituent(s)**	**Sources**	**Family**	**References**
1.	Use of permeation enhancer (s)	Multiple constituents	Multiple sources	Different families	[60]
		Terpenes itself as a permeation enhancers	Multiple sources	Different families	[61]
2.	Encapsulation within a lipid based drug delivery systems	Liposomes	Multiple sources	Different families	[62]
		Solid Lipid Nanoparticles (SLNs) and Nanostructured Lipid Carriers (NLCs)	Multiple sources	Different families	[63,64,65]
3.	Ethosomes	Rutin	Multiple sources	Different families	[66]

**Table 2:** A brief overview regarding Cosmetics and biomedical applications of advanced natural compounds.

Serial No.	Natural Compounds	Cosmetic Applications		Pharmaceutical Applications						
		Skin Whitening	Anti-Oxidant Activity	Anti-Diabetic Activity	Anti-Cancer Efficacy	Anti-Inflammatory Activity	Cardio-Protective Activity or Efficacy Against CVS Disorders	Hepato-Protective Activity or Healing of Liver	Anti-Allergic	Against Endocrine Disorders
1.	Kojic acid	[107]	[108]	[109]	[110]	[111]	[112]	------	------	------
2.	Gallic acid	[113]	[114]	[115]	[116]	[117]	[118]	[119]	[120]	------
3.	Capsaicin	------	[121]	[122]	[123]	[124]	[125]	[126]	------	------
4.	Omega 3 and Omega 6 Fatty acids	[127]	[128]	------	[129]	[130]	[131]	[132]	------	------
5.	Genistein	------	[133]	------	[134]	[135]	[136]	[137]	------	[133]
6.	Carotenoids	------	[138]	[139]	[140]	[141]	[141]	[142]	------	------
7.	Lupeol	------	[143]	[144]	[145]	[146]	------	[147]	------	[148]
8.	Colchicine	[149]	[150]	[151]	------	[152]	[153]	[154]	------	------

## Data Availability

Data sharing not applicable.
